# Roles of Soybean Plasma Membrane Intrinsic Protein GmPIP2;9 in Drought Tolerance and Seed Development

**DOI:** 10.3389/fpls.2018.00530

**Published:** 2018-04-26

**Authors:** Linghong Lu, Changhe Dong, Ruifang Liu, Bin Zhou, Chuang Wang, Huixia Shou

**Affiliations:** ^1^State Key Laboratory of Plant Physiology and Biochemistry, College of Life Sciences, Zhejiang University, Hangzhou, China; ^2^Institute of Horticulture, Zhejiang Academy of Agricultural Sciences, Hangzhou, China; ^3^Institute of Crop Science, Anhui Academy of Agricultural Sciences, Hefei, China; ^4^College of Resources and Environment, Huazhong Agricultural University, Wuhan, China

**Keywords:** aquaporin, drought tolerance, transgenic, soybean, overexpression

## Abstract

Aquaporins play an essential role in water uptake and transport in vascular plants. The soybean genome contains a total of 22 plasma membrane intrinsic protein (PIP) genes. To identify candidate PIPs important for soybean yield and stress tolerance, we studied the transcript levels of all 22 soybean PIPs. We found that a GmPIP2 subfamily member, GmPIP2;9, was predominately expressed in roots and developing seeds. Here, we show that GmPIP2;9 localized to the plasma membrane and had high water channel activity when expressed in *Xenopus* oocytes. Using transgenic soybean plants expressing a native *GmPIP2;9* promoter driving a GUS-reporter gene, it was found high GUS expression in the roots, in particular, in the endoderm, pericycle, and vascular tissues of the roots of transgenic plants. In addition, *GmPIP2;9* was also highly expressed in developing pods. *GmPIP2;9* expression significantly increased in short term of polyethylene glycol (PEG)-mediated drought stress treatment. *GmPIP2;9* overexpression increased tolerance to drought stress in both solution cultures and soil plots. Drought stress in combination with *GmPIP2;9* overexpression increased net CO_2_ assimilation of photosynthesis, stomata conductance, and transpiration rate, suggesting that *GmPIP2;9-*overexpressing transgenic plants were less stressed than wild-type (WT) plants. Furthermore, field experiments showed that *GmPIP2;9*-overexpressing plants had significantly more pod numbers and larger seed sizes than WT plants. In summary, the study demonstrated that GmPIP2;9 has water transport activity. Its relative high expression levels in roots and developing pods are in agreement with the phenotypes of *GmPIP2;9*-overexpressing plants in drought stress tolerance and seed development.

## Introduction

Aquaporins (AQPs) are integral membrane proteins that increase membrane permeability to water and other small molecules (Uehlein et al., 2003; Kaldenhoff and Fischer, 2006; Sade et al., 2010). AQPs play key roles in plant water balance and efficient water use. Plant AQPs are classified into four subfamilies: plasma membrane (PM) intrinsic proteins (PIPs), tonoplast membrane intrinsic proteins (TIPs), nodulin 26-like intrinsic proteins (NIPs), and small basic intrinsic proteins (SIPs)(Luu and Maurel, 2005). PIPs are further divided into two subclasses, PIP1s and PIP2s, based on the length of the *N* terminal in the proteins. It was shown that PIP2s function as water channels when expressed in *Xenopus* oocytes, whereas PIP1s generally have much lower or no water channel activity (Daniels et al., 1994; Kammerloher et al., 1994; Weig et al., 1997; Chaumont et al., 2000; Moshelion et al., 2002). The water permeability of PIP1s requires co-expression of PIP2s to form hetero-tetramers (Chaumont et al., 2000).

Expression of AQPs can be regulated in tissues by hormone treatments and abiotic stresses (Tyerman et al., 2002; Jang et al., 2004; Alexandersson et al., 2005; Chaumont et al., 2005; Bienert et al., 2006; Aroca et al., 2012). In *Arabidopsis*, the expression patterns of AQPs have been evaluated in different organs and under drought stress using microarrays and/or quantitative reverse transcription polymerase chain reaction (qRT-PCR; Jang et al., 2004; Alexandersson et al., 2005, 2010). It was shown that many AQPs are predominantly expressed in either roots or flowers, whereas no AQPs have leaf-specific expression (Alexandersson et al., 2005). With gradual soil drought stress, the abundance of *Arabidopsis* PIPs (*AtPIPs*) transcripts in leaves is generally downregulated, with the exceptions of *AtPIP1;4* and *AtPIP2;5*, which are upregulated, and *AtPIP2;6*, which is unaffected (Alexandersson et al., 2005). Bioinformatic analysis showed strong co-expression of many *AtPIPs* and *AtTIPs* that were downregulated upon drought, whereas *AtPIP1;4*, *AtPIP2;5*, and *AtPIP2;6* are not co-expressed (Alexandersson et al., 2010). Under osmotic stress conditions created by addition of mannitol, many *AtPIPs* are downregulated in aerial parts of the plant, while the transcript levels of *AtPIP1;3*, *AtPIP1;4*, *AtPIP2;1*, and *AtPIP2;5* are upregulated in both root and aerial parts of the mannitol-treated plants (Jang et al., 2004). Studies of rice PIPs (*OsPIPs*) showed that transcription levels of *OsPIP2;1*, *OsPIP2;5*, and *OsPIP2;6* are suppressed in leaves upon polyethylene glycol (PEG)-mediated drought treatment, while *OsPIP1;2* and *OsPIP2;4* are not affected (Guo et al., 2006). In contrast, almost all the *OsPIPs*, especially *OsPIP1;1*, *OsPIP2;5*, and *OsPIP2;7*, were upregulated with short-term PEG treatment in roots.

In addition to water transport in roots, a variety of AQPs are expressed in the coats of developing seeds (Schuurmans et al., 2003). Nutrient and water transport across PMs in seed coats is highly coordinated by regulatory mechanisms and integrates the activities of many nutrient transporters and facilitators (Walker et al., 1995; Zhou et al., 2007a). Thus, it is expected that PIPs that are specifically expressed in native PMs of seed coats are important for seed filling (Zhou et al., 2007a).

Drought is considered one of the most devastating abiotic stress factors that adversely affect crop growth and productivity (Manavalan et al., 2009; Tran and Mochida, 2010). To cope with drought stress, higher plants have evolved sophisticated responses, including stomata closure, increased root/shoot ratio, accumulation of protective solutes and proteins, and production and scavenging of reactive oxygen species (Verslues et al., 2006). Root water uptake can be enhanced or reduced by the overexpression or loss of one or more PIP genes, respectively (Aharon et al., 2003; Javot, 2003; Zhou et al., 2014). These findings suggest that alteration of the expression of certain PIPs in transgenic crops can improve the tolerance to drought stress.

A recent study showed that altered plant transpiration led to rapid changes in root expression of soybean *PIP1;6* (*GmPIP1;6*) that correlated with changes in root hydraulic conductance (Vandeleur et al., 2014). Thus, GmPIP1;6 is proposed to play a role in regulating root hydraulic conductance. Consistent with this idea, our previous study showed that under salt stress, overexpression of *GmPIP1;6* enhanced root water transport, photosynthesis, and seed filling (Zhou et al., 2014). The soybean genome contains a total of 22 PIP genes (Sakurai et al., 2005). To identify candidate *PIPs* important for soybean yield and stress tolerance, we studied the transcript levels of all 22 soybean *PIPs*. We found that a GmPIP2 subfamily member, *GmPIP2;9*, was predominately expressed in roots and developing seeds. In this study, we developed and characterized transgenic soybean plants that overexpressed *GmPIP2;9*. Our results showed that *GmPIP2;9* overexpression conferred enhanced drought stress tolerance and improved seed setting and filling.

## Materials and Methods

### Plant Materials, Growth Conditions, and Treatments

We used soybean cultivar Williams 82 for all physiological experiments and as the recipient for transformations. For physiological experiments, we germinated seeds in filter papers for 4 days prior to transferring into half-strength Hoagland’s solution (Hoagland and Arnon, 1950) or soil pots. After germination, we grew soybean seedlings in growth chambers with a 12-h photoperiod (200 μmol photons m^-2^ s^-1^) and a day/night temperature of 30/22°C.

For PEG-mediated drought treatments, we transferred 10-day-old seedlings into half-strength Hoagland’s solution with or without addition of 20% PEG 8000. For the soil pot drought tolerance test, soybean seeds were planted in a soil pot for 2 weeks. The soil pots in the drought treatment were not watered for 12 days, followed by re-watering for 3 days.

In a different soil pot experiment, 40-day-old plants were not watered for 21 days and then re-watered for 7 days. The transgenic and wild-type (WT) plants were grown together in pots to minimize experimental error. We used five pots for each treatment group, including the control. Soil water content during the treatment was recorded daily (Supplementary Figure S1).

### Quantitative and Semi-quantitative RT-PCR

We measured *GmPIP2;9* expression in different tissues and during drought treatment with qRT-PCR. Samples for tissue-specific expression analysis were collected from three different plants as three biological replicates. RNA extraction and reverse transcription were performed using TRIzol reagent and SuperScript II reverse transcriptase, respectively (Invitrogen, Carlsbad, CA, United States). We performed qRT-PCR performed using SYBR Green I dye as a fluorescent signal. We calculated relative expression levels for three biological replicates using the 2^-ΔΔ*C*_t_^ method. The primer pair for the qRT-PCR of *GmPIP2;9* was 5′-TCACTTGGCAACCATCCCAG-3′ and 5′-CAAGAGCCTTAGCAGCACCT-3′. The primer pair used for the housekeeping gene *GmACTIN* was 5′-CAGAGAAAGTGCCCAAATCATGT-3′ and 5′-TTGCATACAAGGAGAGAACAGCTT-3′.

### Construction of Vectors and Soybean Transformation

We constructed binary vectors for overexpression of *GmPIP2;9* and expression of *GmPIP2;9* promoter-fused β-glucuronidase (GUS)-reporter (P*_PIP2;9_*::GUS) as follows: we amplified full-length 858 bp *GmPIP2;9* cDNA (Phytozome No. Glyma.02g073600) by RT-PCR, using the primers 5′-GCTCTAGAATGGCTAAGCATGATGTTGAG-3′ and 5′-CGGGATCCTCAAATAGTGGGGTTGCTCCT-3′. Then, we cloned the amplified fragment into the pTF101.1-derived vector pLM-B001 (Paz et al., 2004) under control of the 35S promoter of the cauliflower mosaic virus. For the P*_PIP2;9_*::GUS construct, we amplified the promoter region of a 2-kb fragment upstream of the ATG start codon from Williams 82 genomic DNA, using the primers 5′-CGGGATCCGTGTTTTATCACATATACACACATTTT-3′ and 5′-GCTCTAGATGCAATTTGCAACTACCCTTT-3′. The PCR product was cloned into the pTF101.1-derived vector PTF101-GUS. The resulting vectors were confirmed by sequencing and transfected into *Agrobacterium tumefaciens* strain EHA101. We performed transformations of vectors into soybean as previously described (Song et al., 2013).

### GUS Histochemical Analysis

We took samples of leaves, roots, flowers, and pods from P*_PIP2;9_*::GUS transgenic soybean plants and stained them with GUS histochemical staining buffer, containing 100 mM sodium phosphate buffer (pH 7.0), 1 mM 5-bromo-4-chloro-3-indolyl-b-D-glucuronidase (X-Gluc), 1 mM K_4_[Fe(CN)_6_], 1 mM K_3_[Fe(CN)_6_], 0.5% (v/v) Triton X-100, and 20% (v/v) methanol. To prepare the root sections, we embedded soybean roots using 5% (w/v) low-melting-point agarose (Sigma, St Louis, MO, United States) and cut sections with a vibrating blade microtome (Leica VT1000S; Heidelberger, Germany). We examined all samples under a bright-field a microscope (Nikon, Tokyo, Japan).

### Subcellular Localization of GmPIP2;9 in Plant Cells

We amplified full-length *GmPIP2;9* cDNA without a stop code, using the primers 5′-TGTCGGAGCTCGGTACCCATGGCTAAGCATGATGTTGAGGGTG-3′ and 5′-TCGCCCTTGCTCACCATGTCCAATAGTGGGGTTGCTCCTGAAT-3′. The PCR product was fused to the 5′ end of the green fluorescent protein (GFP)-encoding gene under control of the CaMV 35S promoter in the pCAMBIA1302 vector^1^. We used a construct carrying AtPIP2A::mCherry as a marker for PM localization (Nelson et al., 2007). We transiently co-expressed the two constructs in onion epidermal cells, as previously described (Zhou et al., 2014).

### Subcellular Localization and Water-Permeability Assay of GmPIP2;9 in *Xenopus* Oocytes

To analyze the water transport activity of GmPIP2;9, we ligated the amplified fragment of the *GmPIP2;9* cDNA or nYFP-fused GmPIP2;9 sequence into the *Xenopus* oocyte expression vector pGEMHE (Liman et al., 1992). The pGEMHE::nYFP-GmPIP2;9 was injected into oocytes and incubated for 48 h in BS before imaging with confocal laser scanning microscopy to determine the subcellular localization of GmPIP2;9 in oocytes. The pGEMHE::GmPIP2;9 vector was transcribed into complementary RNA (cRNA), as previously described (Vandeleur et al., 2009). We injected 23 ng of cRNA into oocyte cells of *Xenopus laevis* using a Nanoject microinjector (Drummond Scientific Co., Broomall, PA, United States). *Xenopus* oocytes were incubated in Ca-Ringer’s solution with horse serum and antibiotics at 18°C for 1 day and then transferred the oocytes into a 5× diluted ND96 solution. We measured and analyzed oocyte volume, as previously described (Fetter et al., 2004; Vandeleur et al., 2009). Osmotic water permeability (*P*_os_) was calculated using the equation *P*_os_ = *J*_w_/*V*_w_ × *A* × Δ*O*_sm_, where *J*_w_ = the initial rate of change of relative cell volume, *V*_w_ = the partial molar volume of water, *A* = the area of the oocyte, and Δ*O*_sm_ = the change in osmolarity.

### Measurement of Net CO_2_ Assimilation (A*_N_*), Stomatal Conductance (g*_s_*), and Transpiration Rate (T*_r_*)

We grew soybean plants in soil pots with a normal water and nutrient supply for 40 days in a greenhouse prior to drought treatment. We recorded the *A_N_*, *g_s_*, and *T_r_* of fully expanded leaves using an Li-6400 portable gas-exchange system (LI-COR, NE, United States) on day 11 of the drought treatment. We performed all measurements between 8:00 AM and 2:00 PM. The photosynthetic photon flux density was 1200 μmolm^-2^ s^-1^, the leaf surface temperature was 30°C, and the CO_2_ concentration was 400 μmol mol^-1^. To minimize experimental error, we planted *GmPIP2;9*-Oe plants and WT plants together as shown in **Figure 6**.

### Agronomic Performance of WT and GmPIP2;9-Oe Transgenic Plants Grown in the Field

We measured agronomic characteristics, including plant height, numbers of branches, nodes, pods, and seeds per plant, 100-seed weight, and seed weight per plant using samples from a 2015 field experiment at the Anhui Academy of Agricultural Sciences. The field experiment was arranged in a triplicate randomized block design with 10 m^2^ for each plot. For each replication, we sampled 10 plants from each of two transgenic (*GmPIP2;9*-Oe1 and Oe2) lines and the WT line.

### Statistical Analysis

Statistical analysis of the data was performed using the Data Processing System (DPS version 7.05, Zhejiang, China). We used Student’s *t*-tests to determine significant differences between the WT and transgenic lines for each treatment. We used least significant difference tests for pairwise comparisons between samples.

## Results

### GmPIP2;9 Localizes to the PM

**FIGURE 1:**
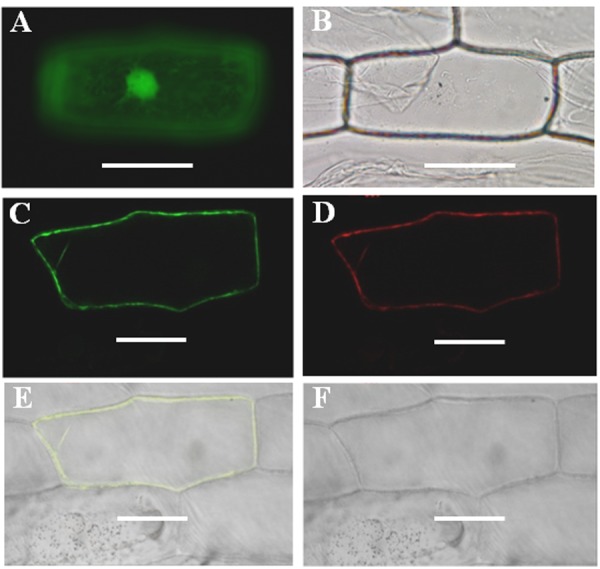
Subcellular localization of GmPIP2;9. **(A,B)** Onion epidermal cells expressing green fluorescent protein (GFP). **(A)** GFP signals in the nucleus, cytoplasm, and plasma membrane (PM). **(B)** Bright-field light image. **(C)** Onion epidermal cells expressing GmPIP2;9-GFP with GFP signals in the PM. **(D)** Onion epidermal cells expressing red fluorescent signal from the CD3-1007 PM marker. **(E,F)** Merged fluorescent and bright-field images of an epidermal cell expressing the GmPIP2;9-sGFP fusion protein and the CD3-1007 PM marker. Bars = 100 μm.

GmPIP2;9 is predicted to localize to the PM (Zhou et al., 2013). To obtain direct experimental evidence, we transiently co-expressed a 35S-GmPIP2;9::GFP construct and a mCherry-fused PM marker CD3-1007 in onion epidermal cells. As shown in **Figure 1**, 35S-GmPIP2;9::GFP fluorescence was confined to the PM and co-localized with CD3-1007 red fluorescence. In contrast, the green fluorescent signal from the control construct with GFP alone was distributed throughout the nucleus and cytoplasm.

### GmPIP2;9 Has High Water Channel Activity When Expressed in *Xenopus* Oocytes

**FIGURE 2:**
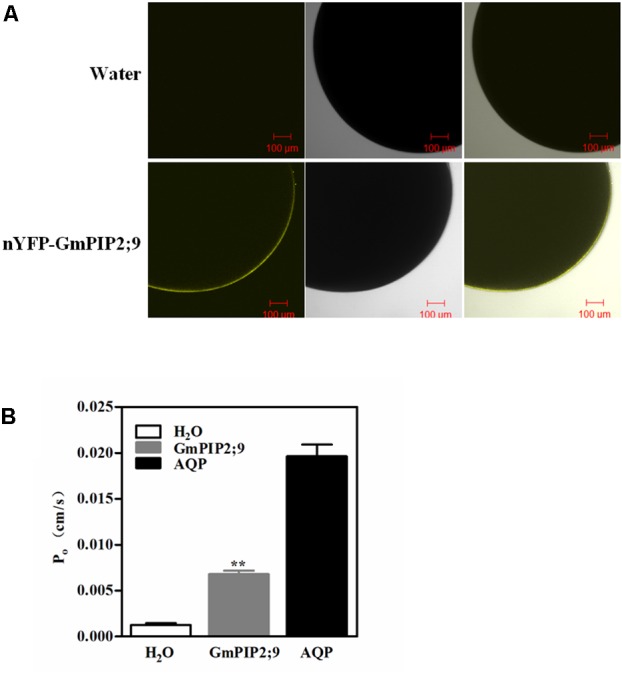
Expression of functional *GmPIP2;9* in *Xenopus* oocytes. **(A)** YFP localization of nYFP-GmPIP2;9 in *Xenopus* oocytes. The oocytes were observed 2 days after injection as described in Section “Methods.” Bars = 100 μ m. Fluorescence (left), bright-field (middle), and merged images (right) showing that nYFP-GmPIP2;9 fusion protein is expressed in plasma membrane of oocytes. **(B)** Oocytes were injected with 23 ng *GmPIP2;9* cRNA or water. The osmotic water permeability coefficients (*P*_f_) of oocytes were determined from swelling kinetics. Data represent the mean ± *SD* measurements from 10 oocytes. ^∗∗^*P* < 0.01.

Plant PIP2 proteins that have been examined so far have high water channel activity when expressed in *Xenopus* oocytes (Fetter et al., 2004; Verdoucq et al., 2008). To confirm whether GmPIP2;9 has water channel activity, we first investigated the localization of GmPIP2;9 in *Xenopus* oocytes. As shown in **Figure 2A**, expression of pGEMHE::nYFP-GmPIP2;9 construct into *Xenopus* oocytes resulted in PM localization of the YFP-GmPIP2;9 fusion protein. The osmotic water permeability coefficient (*P*_f_) was measured by injected with *GmPIP2;9* cRNA. We used human AQP cRNA (*AQP*) and water as the positive and negative controls, respectively. We found a significantly higher *P*_f_ value in oocytes injected with *GmPIP2;9* than in oocytes injected with the water control (**Figure 2B**), suggesting that *GmPIP2;9* is a functional AQP with high water channel activity in *Xenopus* oocytes. The high water channel activity of GmPIP2;9 was confirmed in a separate oocyte expression experiment that compared the *P*_f_ values of nYFP-GmPIP1;6 (Zhou et al., 2014), nYFP-GmPIP2;9, and nYFP-GmPIP1;6+GmPIP2;9 cRNA (Supplementary Figure S2). While nYFP-GmPIP1;6 had no water channel activity, nYFP-GmPIP2;9 showed a similar *Pf* value to GmPIP2;9 (Supplementary Figure S2).

### Expression Patterns of GmPIP2;9 in Different Tissues and in Response to Osmotic Stress

**FIGURE 3:**
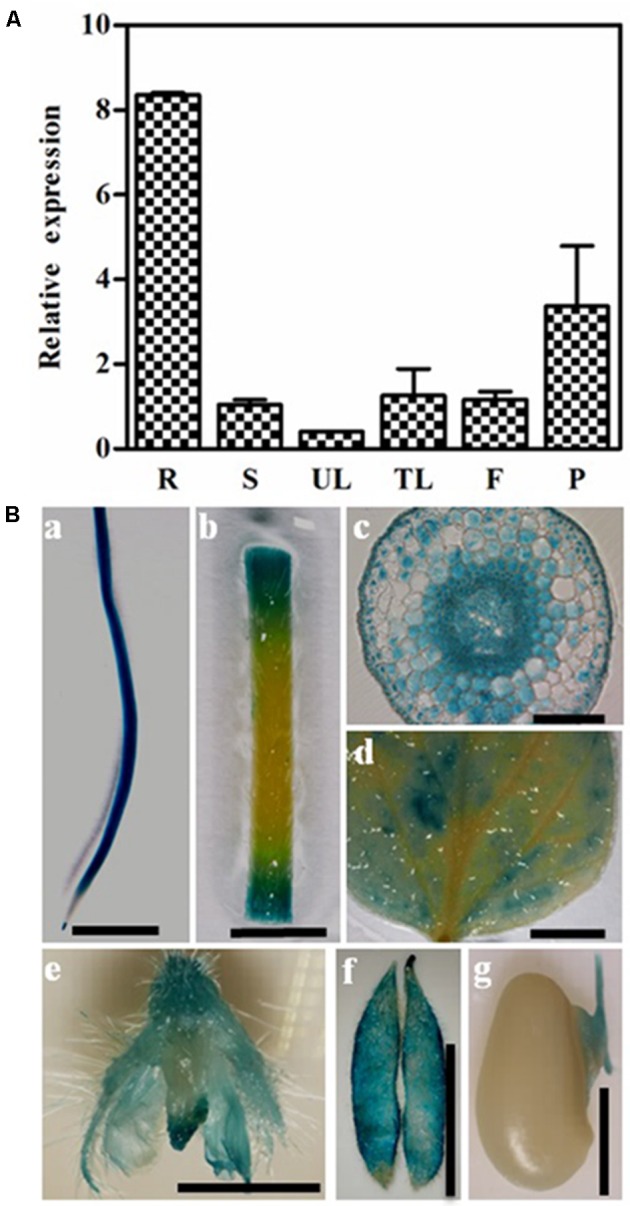
Expression patterns of *GmPIP2;9*. **(A)** Relative expression levels of *GmPIP2;9* in root (R), stem (S), unifoliate leaf (UL), trifoliolate leaf (TL), flower (F), and pod (P). All data are means of three biological replicates with error bars indicating *SD*. **(B)** β-glucuronidase (GUS) staining of root **(a)**, stem **(b)**, cross section of root **(c)**, leaf **(d)**, flower **(e)**, pod **(f)**, and developing seeds **(g)**. Bar = 1 cm in **a**, **b**, **d**, **e**, and **f**. Bar = 1 mm in **c** and **g**.

We used qRT-PCR to examine the expression of *GmPIP2;9* in different soybean tissues and found that *GmPIP2;9* was expressed in all examined tissues, with the highest expression in roots (**Figure 3A**). Furthermore, using transgenic soybean plants expressing a native *GmPIP2;9* promoter driving a GUS-reporter gene, we confirmed that *GmPIP2;9* was expressed in all examined tissues, including roots, leaves, stems, flowers, and pods during the seed development stage (**Figure 3B**). Consistent with qRT-PCR results, we observed high GUS expression in the roots of transgenic plants (**Figure 3B**). Cross sections of root tissues showed that *GmPIP2;9* was predominantly expressed in the endoderm, pericycle, and vascular tissues (**Figure 3B**). Notably, we observed high GUS expression in the pods and the seed hilum, which assimilate and transport water.

**FIGURE 4:**
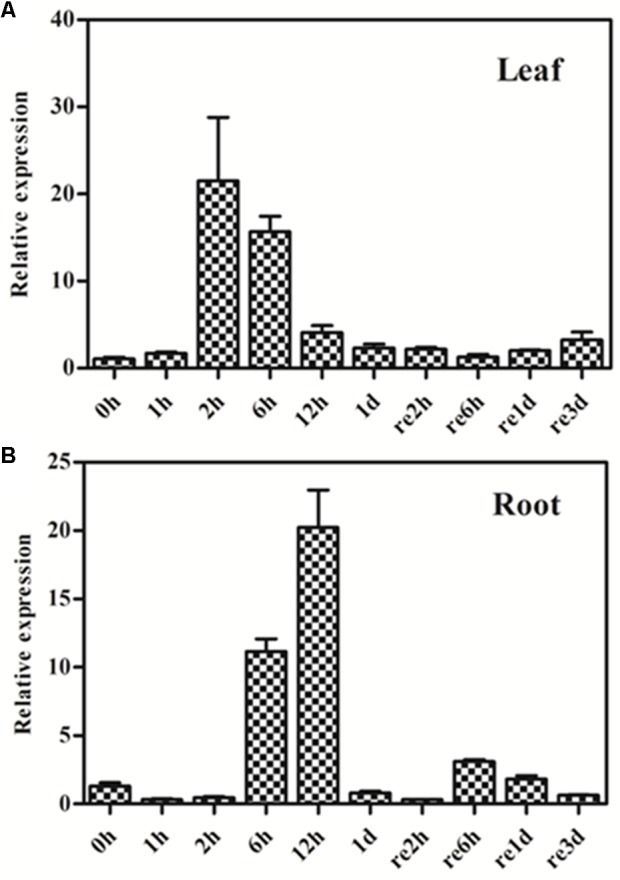
Expression pattern of *GmPIP2;9* in soybean plants under normal and drought treatments. Thirteen-day-old soybean seedlings were treated with or without 20% polyethylene glycol (PEG) in nutrient solution. Total RNA was extracted from **(A)** leaves and **(B)** roots of these seedlings at 1 h, 2 h, 6 h, 12 h, and 1 day (1–12h and 1d) of PEG treatment, and 2 h, 6 h, 1 day, and 3 days of the recovery (re2h, re6h, re1d, and re3d). Data represent the means ± *SD*. Three replicates were used. Expression levels of treated plants are relative to control plants at each time point.

We also investigated the effect of osmotic stress on *GmPIP2;9* expression, using a 20% PEG drought treatment in 13-day-old seedlings. We found increased *GmPIP2;9* expression in roots after 6 h of PEG treatment and expression was highest after 12 h of the treatment (**Figure 4B**). In roots, *GmPIP2;9* expression levels returned to baseline within 24 h, although the osmotic stress was not removed (**Figure 4B**). In leaves, *GmPIP2;9* expression increased within 2 h of PEG treatment, which was faster than in roots (**Figure 4A**). Expression returned to baseline within 1 day also without recovery (**Figure 4A**). These results suggest that GmPIP2;9 responds quickly to osmotic stress.

### Generation of Transgenic Soybean Plants Overexpressing GmPIP2;9

To further investigate the function of *GmPIP2;9* in drought stress tolerance, we generated transgenic soybean plants that overexpressed *GmPIP2;9* (*GmPIP2;9*-Oe). The T-DNA region of the binary vector used for soybean transformation is shown in Supplementary Figure S3A. We obtained five independent transgenic events and verified these by semi-quantitative RT-PCR (Supplementary Figure S3B). We found that the abundance of *GmPIP2;9* transcripts in the leaves and seeds of the T1 plants from the Oe1 and Oe2 events was more than 20-fold higher than in WT leaves and seeds (Supplementary Figure S3C). Thus, we used these two events (*GmPIP2;9*-Oe1 and *GmPIP2;9*-Oe2) for further experiments.

### Overexpression of GmPIP2;9 Enhances Drought Tolerance

**FIGURE 5:**
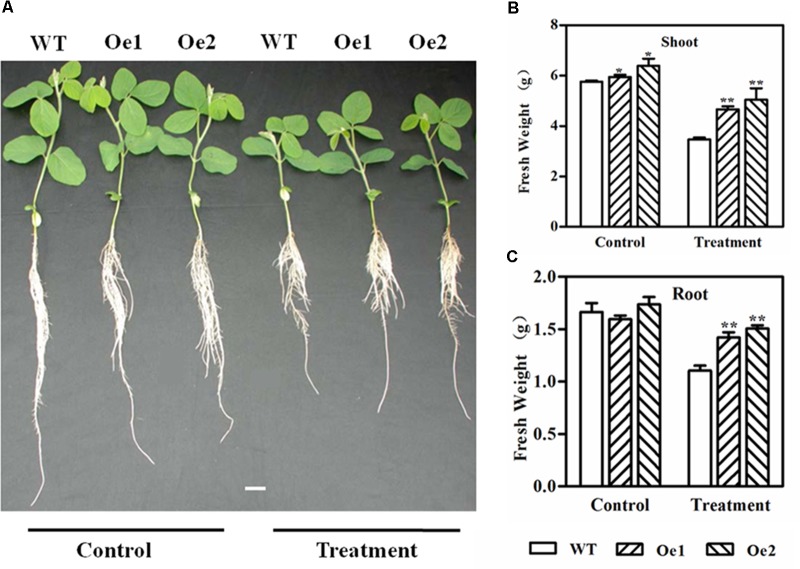
Growth performance of wild-type (WT) and *GmPIP2;9*-overexpression (Oe) plants under normal and polyethylene glycol (PEG)-mediated drought conditions. **(A)** Thirteen-day-old WT and *GmPIP2;9*-Oe plants were treated with or without 20% PEG in culture media for 2 days, followed by a 5-day recovery. **(B)** Fresh shoot weight. **(C)** Fresh root weight. Data are means ± *SD* (*n* = 3). ^∗^*P* < 0.05 and ^∗∗^*P* < 0.01.

**FIGURE 6:**
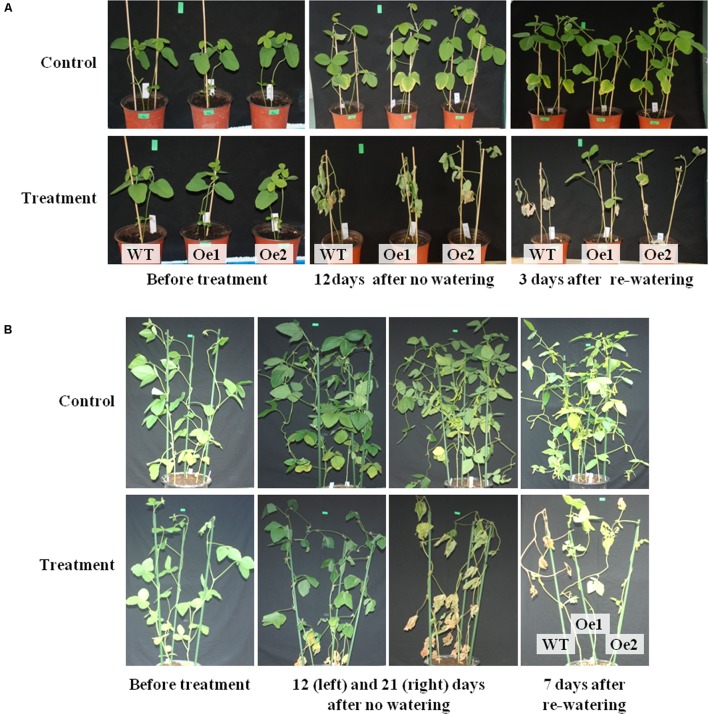
Performance of wild-type (WT) and *GmPIP2;9*-overexpression (Oe) plants under drought stress conditions. **(A)** Drought stress treatment was applied to 2-week-old seedlings of WT, *GmPIP2;9*-Oe1, and -Oe2 lines by withholding water for 12 days and then re-watering for 3 days. **(B)** Water was withheld from 40-day-old WT and *GmPIP2;9*-Oe plants for 21 days, and then plants were re-watered for 7 days.

To investigate the role of *GmPIP2;9* in drought tolerance, we treated 13-day-old *GmPIP2;9*-Oe1, *GmPIP2;9*-Oe2, and WT plants with 20% PEG in hydroponic solution cultures for 2 days, followed by a 5-day recovery. As shown in **Figure 5A**, PEG treatment suppressed the growth of both WT and transgenic plants. Although the stress-treated plants were significantly smaller than the non-stressed plants for both the WT and transgenic plants, the fresh weights of aerial parts and roots of PEG-treated *GmPIP2;9*-Oe1 and -Oe2 plants were significantly higher than that of the WT plants (**Figures 5B**, **6C**). These results indicate that overexpression of *GmPIP2;9* in soybean enhances tolerance to osmotic stress.

To further assess the effect of *GmPIP2;9* overexpression on drought tolerance, we withheld water from 2-week-old plants in soil pots to apply drought stress. The drought treatment was applied for 12 days, after which the stressed plants were completely wilted. When the wilted plants were re-watered, the *GmPIP2;9*-Oe plants revived, but WT plants did not recover (**Figure 6A**).

In an additional drought tolerance experiment, we withheld water from 40-day-old, pot-grown *GmPIP2;9*-Oe1, *GmPIP2;9*-Oe2, and WT plants for 3 weeks, followed by re-watering for 1 week. After 12 days without watering, the leaves of WT and *GmPIP2;9-Oe* plants began to wilt (**Figure 6B**). After 3 weeks without watering, the leaves of the WT and *GmPIP2;9-Oe* lines were completely wilted (**Figure 6B**). Re-watering revived the two transgenic plant lines, but WT plants did not recover (**Figure 6B**).

### GmPIP2;9-Oe Plants Maintain High Photosynthesis Rates Under Drought Stress

**FIGURE 7:**
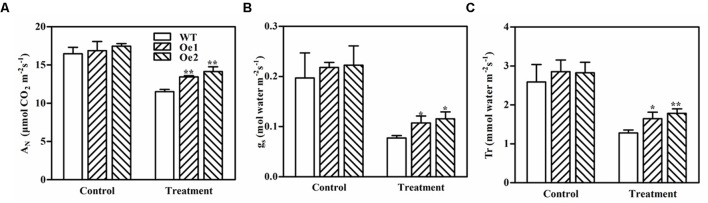
Daily **(A)** net CO_2_ assimilation (*A_N_*), **(B)** stomatal conductance (*g_s_*), and **(C)** transpiration rate (*T_r_*) of wild-type (WT) and *GmPIP2;9*-overexpression (Oe) transgenic soybean plants under normal and drought stress conditions. Water was withheld from 40-day-old WT and *GmPIP2;9*-Oe transgenic soybean plants for 11 days. An LI-6400 was used to measure *A_N_*, *g_s_*, and *T_r_* between 8 AM and 2 PM on day 11 of drought treatment. The parameters were measured in four different plants per treatment. Data are the means ± *SD* (*n* = 4). ^∗^*P* < 0.05 and ^∗∗^*P* < 0.01.

We measured photosynthetic processes, including net CO2 assimilation (*A_N_*), stomatal conductance (*g_s_*), and transpiration rate (*T_r_*), in the leaves of *GmPIP2;9*-Oe1, *GmPIP2;9*-Oe2, and WT plants after 11 days of drought treatment (**Figure 7**). Although drought stress significantly suppressed these photosynthetic processes in all plants, the *GmPIP2;9*-Oe1 and -Oe2 plants maintained significantly higher photosynthetic rates than WT plants (**Figures 7A–C**). These results further suggest that GmPIP2;9 plays a role in drought tolerance.

### Overexpression of GmPIP2;9 Results in Increased Seed Number and Seed Size in the Field

We grew WT plants and homozygous transgenic T3 plants from two independent *GmPIP2;9*-Oe transformation events and evaluated their agronomic traits under field conditions. We found a significantly higher pod number, seed number, and seed weight per plant in *GmPIP2;9*-Oe plants than in WT plants (**Table 1** and Supplementary Figure S4).

**Table 1:** Agronomic performance of wild-type (WT) and GmPIP2;9-overexpre- ssion (Oe) plants.

Traits	WT	Oe1	Oe2
Plant height (cm)	48.8 ± 4.6b	59.1 ± 6.4a	57.8 ± 3.8a
Number of branches	2.7 ± 0.5b	3.5 ± 1.1a	4.1 ± 1.0a
Number of nodes/plant	16 ± 1.7b	17.4 ± 2.2a	17.7 ± 0.7a
Number of pods/plant	71 ± 22.3b	92.8 ± 27.5a	94.3 ± 20.2a
Number of seeds/plant	168.7 ± 55.2b	219.5 ± 57.8a	220 ± 45.7a
Seed weight (g)/plant	26.3 ± 7.8b	34.9 ± 9.7a	33.6 ± 6.8a
100-seed weight (g)	15.4 ± 1.1b	17.0 ± 1.0a	16.9 ± 0.7a


*Data are given as means ± *SD* (*n* = 30). Different letters indicate significant differences (least significant difference test, *P* < 0.05).*

## Discussion

Regulation of water transport is crucial for drought tolerance in crop plants, such as soybeans. PIPs are involved in water transport and predicted to localize to the PM (Zhou et al., 2013). There are eight *PIP1* and 14 *PIP2* genes in the soybean genome; however, the specific functions of individual PIP proteins are largely unknown. Thus, we have a limited understanding of how water transport is regulated within plants and from the outside environment. Here, we confirmed that GmPIP2;9 has functional water channel activity when expressed in *Xenopus* oocytes. We also developed transgenic soybean lines that overexpress *GmPIP2;9*. GmPIP2;9 overexpression significantly increased drought stress tolerance (**Figures 5**, **6**) and the transgenic plants had significantly increased seed number and seed size, indicating enhanced yields (**Table 1** and Supplementary Figure S4).

Analysis of transgenic plants expressing a P*_PIP2;9_*::GUS reporter construct revealed that *GmPIP2;9* is predominantly expressed in roots (**Figure 3Ba–g**). High expression in roots suggests that GmPIP2;9 plays an important role in water transport in soybean roots. Given this finding, we expected that GmPIP2;9 overexpression would increase water flow across roots and enhance tolerance to drought stress. This hypothesis was supported by the finding that the *GmPIP2;9*-Oe1 and *GmPIP2;9*-Oe2 transgenic plants were less susceptible to drought stress and recovered from drought stress when WT plants did not (**Figure 6**).

Developing legume seeds import nutrients by mass flow through the phloem (Zhou et al., 2007a,b). To maintain water balance, the import of nutrients via phloem and the transport of water through pod walls and seed coats are maintained at similar levels (Zhou et al., 2007a,b). The high expression of *GmPIP2;9* in the developing pod and seed hilum, which we observed in *GmPIP2;9* promoter::GUS transgenic plants (**Figure 3**), suggests that GmPIP2;9 may facilitate water transport through pod walls and from seed coats to developing seeds. Consequently, seed abortion rates in *GmPIP2;9*-Oe plants were reduced, as evidenced by the increased seed numbers per plant. Furthermore, we saw that *GmPIP2;9*-Oe seeds were more filled than WT seeds, as evidenced by increased seed weights (**Table 1**). Additionally, strong GUS expression in pods and hilum of the developing seeds in *GmPIP2;9* promoter::GUS transgenic plants supports our hypothesis.

This study, combined with previous work, has indicated several avenues for additional investigation to further define the role of GmPIP2;9 in water transport. It was reported that the expression and the transport activity of many plant AQPs are regulated at transcriptional and post-translational level (Liu et al., 2013). Studies of *GmPIP2;9* expression in response to hormones, such as GA3, ABA, and brassinolide, should bring us information about what signaling pathways GmPIP2;9 is associated with. Furthermore, developing antibody to directly examine the levels of GmPIP2;9 proteins in different soybean tissues and under stress conditions may clarify the exact physiological mechanism by which overexpression of *GmPIP2;9* increased the tolerance to drought stress. Finally, comparison of water transport activities in native PMs of soybean seed coats from *GmPIP2;9*-Oe transgenic and WT plants, and development of *GmPIP2;9* knocking out materials using the CRISPR/CAS9 system will bring additional insights to understand the roles of GmPIP2;9.

## Conclusion

We developed transgenic soybean plants that expressed 35S-GmPIP2;9 or *GmPIP2;9* promoter::GUS reporter constructs. We demonstrated that: (1) GmPIP2;9-Oe plants have increased drought tolerance and this enhanced drought tolerance is consistent with the predominant expression of the gene in roots and (2) GmPIP2;9-Oe plants have increased pod number, seed number, and seed weight, indicating higher yields. It may associate with the relative high expression of *GmPIP2;9* in developing seeds, or by enhanced water uptake from the roots which indirectly improved photosynthetic efficiency.

## Author Contributions

HS and CW designed the experiments. LL, CD, RL, BZ, and CW conducted the experiments and analyzed the data. LL, CD, and HS wrote the manuscript. All authors read and approved the manuscript.

## Conflict of Interest Statement

The authors declare that the research was conducted in the absence of any commercial or financial relationships that could be construed as a potential conflict of interest. The reviewer JY and handling Editor declared their shared affiliation.

## Footnotes

1 http://www.cambia.org/daisy/cambia/585.html#dsy585_Description
