# Supplementary figures and images for: Roles of Soybean Plasma Membrane Intrinsic Protein GmPIP2;9 in Drought Tolerance and Seed Development
Source: Front Plant Sci. 2018 Apr 26;9:530. doi: 10.3389/fpls.2018.00530 (PMC5932197; doi:10.3389/fpls.2018.00530)

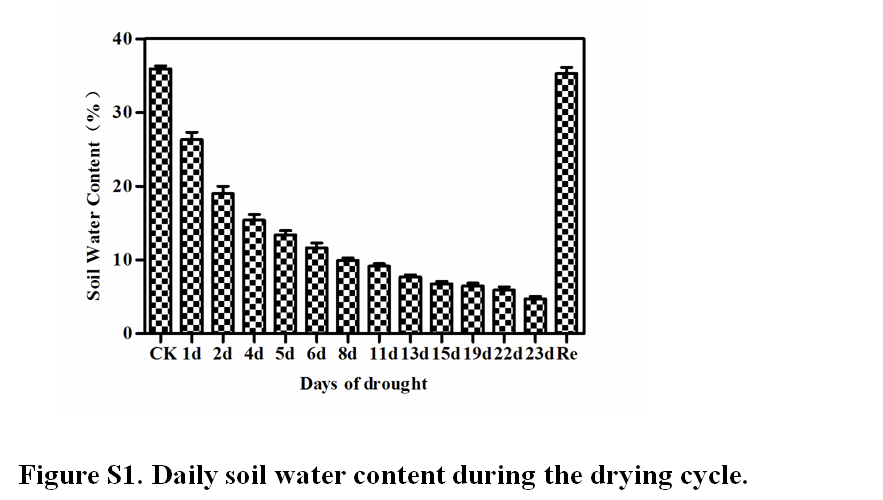

Supplement: Supplementary file 1 [file Image_1.TIF]

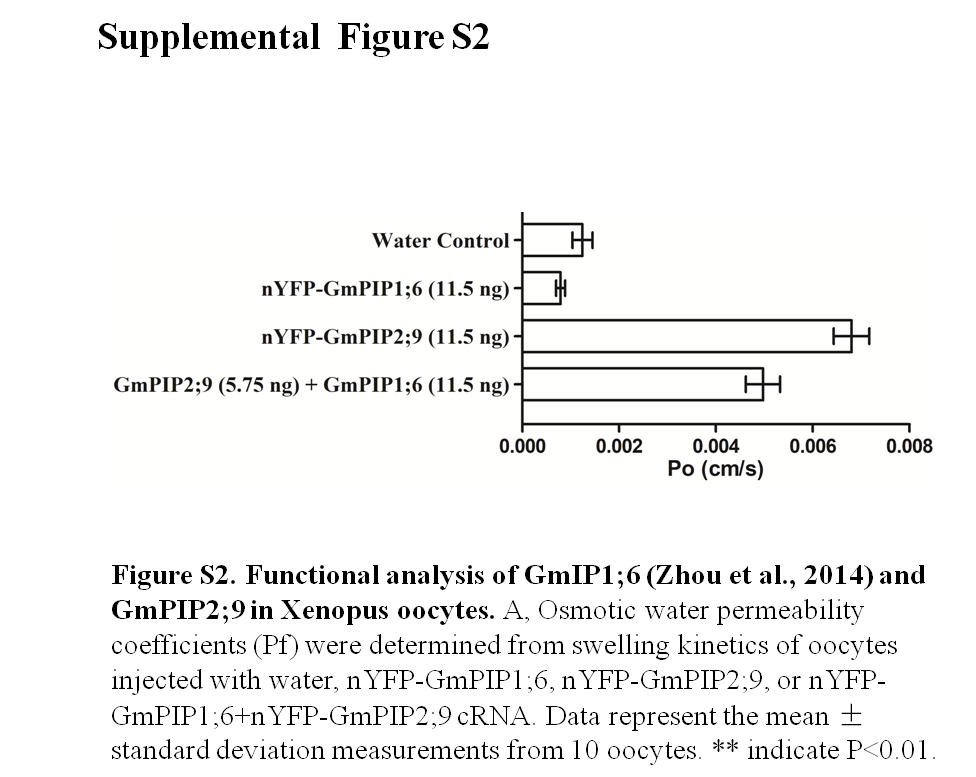

Supplement: Supplementary file 2 [file Image_2.TIF]

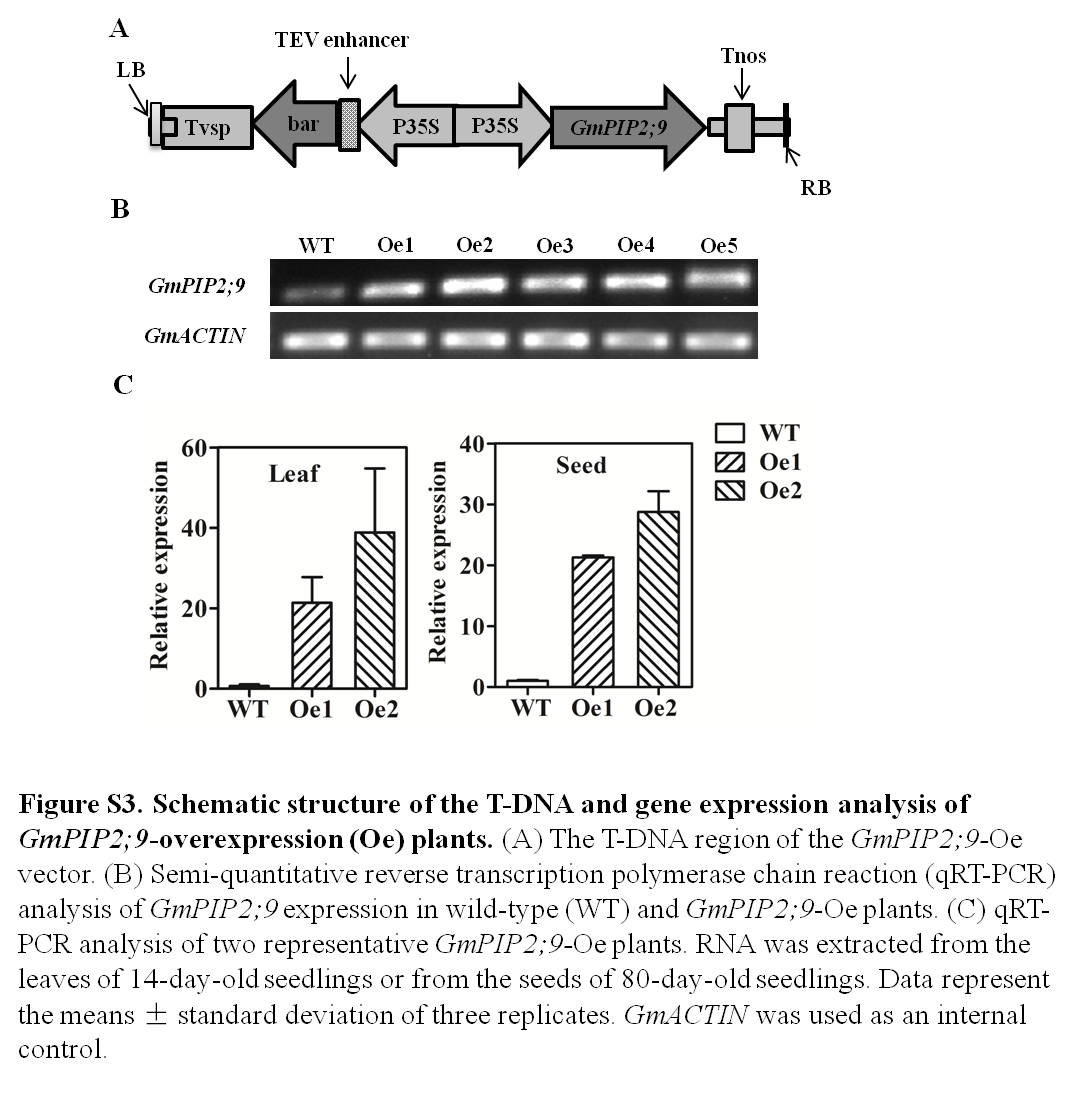

Supplement: Supplementary file 3 [file Image_3.TIF]

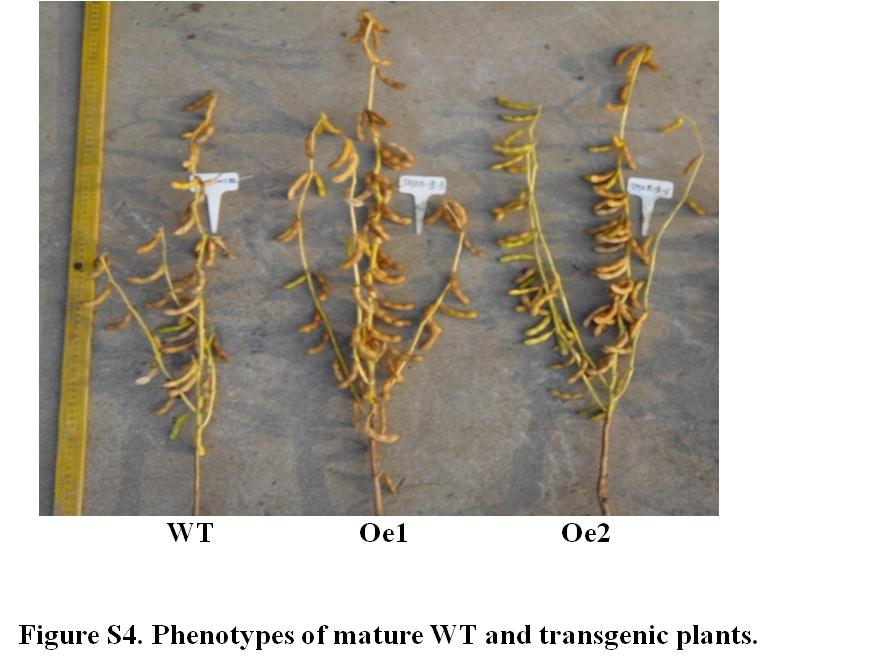

Supplement: Supplementary file 4 [file Image_4.TIF]
